# Radiation De-Escalation in Older Women with Early-Stage ER+/HER2– Invasive Breast Cancer

**DOI:** 10.1245/s10434-025-18746-z

**Published:** 2025-11-23

**Authors:** Ton Wang, Drew Neish, Samantha Thomas, Astrid Botty van den Bruele, Laura H. Rosenberger, Akiko Chiba, Kendra Parrish, Lesly A. Dossett, Gayle DiLalla, Jennifer K. Plichta, Maggie L. DiNome, E. Shelley Hwang, Diandra Ayala-Peacock

**Affiliations:** 1 Department of Surgery, Duke University Medical Center, Durham, NC USA; 2 Duke Cancer Institute, Duke University, Durham, NC USA; 3 Biostatistics and Bioinformatics, Duke University, Durham, NC USA; 4 Department of Surgery, Michigan Medicine, Ann Arbor, MI USA; 5 Department of Population Health Sciences, Duke University Medical Center, Durham, NC USA; 6 Department of Radiation Oncology, Duke University Medical Center, Durham, NC USA

**Keywords:** Radiation omission, Breast cancer, De-escalation, Hypofractionation, Abbreviated radiotherapy

## Abstract

**Background:**

National recommendations since 2004 have allowed for omission of post-lumpectomy radiotherapy for patients ≥70 years old with early-stage estrogen receptor positive (ER+)/human epidermal growth factor receptor 2-negative (HER2–) breast cancer. Rates of omission in the era of abbreviated radiotherapy regimens are unknown. This study aimed (1) to determine contemporary trends in post-lumpectomy radiotherapy omission, (2) to determine trends in use of partial-breast (PB), ultra-hypofractionated (UHF), moderately hypofractionated (MHF), and conventionally fractionated (CF) radiotherapy regimens, and (3) to identify targetable factors to de-escalate radiotherapy in patients eligible for omission.

**Methods:**

A retrospective cohort analysis was performed using the National Cancer Database. The study identified patients ≥70 years old with pT1, cN0 pNX-0, cM0 ER+/HER2– breast cancer who underwent lumpectomy from 2012 to 2021. Radiotherapy treatment strategies based on number of fractions received were evaluated and compared using multivariable analysis.

**Results:**

The study included 121,160 patients: 51.0 % with no radiotherapy, 8.4 % with PB, 4.1 % with UHF, 30.2 % with MHF, and 6.3 % with CF radiotherapy. Radiotherapy omission decreased from 53.7 % to 46.8 % between 2012 and 2021. Whereas MHF radiotherapy doubled from 16.4 % to 32.9 %, CF radiotherapy decreased from 17.1 % to 2.0 %. A minority of patients received PB or UHF radiotherapy, although the rate of UHF increased from 4.6 % to 7.7 % between 2020 and 2021. Treatment at academic/research or Integrated Network Cancer Program facilities and higher-volume hospitals was associated with increased likelihood of treatment with abbreviated radiotherapy regimens.

**Conclusions:**

Despite efforts to promote treatment de-escalation, rates for omission of post-lumpectomy radiotherapy have decreased over time. To avoid overtreatment, patients who are eligible for omission but opt to receive radiotherapy should be considered for PB or UHF radiotherapy regimens.

**Supplementary Information:**

The online version contains supplementary material available at 10.1245/s10434-025-18746-z.

Approximately one third of new breast cancers (BC) occur in women ≥70 years old.^1^ The majority of these patients have a diagnosis of early-stage, estrogen receptor-positive (ER+)/human epidermal growth factor receptor 2 (HER2)-neu receptor-negative (HER2–) invasive BC, which is associated with a 10-year disease-specific survival rate higher than 98 %.^2^ Given this excellent prognosis, there have been substantial efforts to safely de-escalate BC treatment for older adult patients. As a prominent example, current national guidelines allow for omission of adjuvant radiotherapy after lumpectomy in women ≥70 years old with pathologic T1 (pT1), clinically node-negative (cN0) BC who receive endocrine therapy.^3^ This recommendation is based on the CALGB 9343 and PRIME II randomized clinical trials, which showed that although adjuvant radiotherapy after breast conservation for these patients was associated with an improvement in rates of locoregional recurrence, it did not improve overall or distant disease-free survival.^2,4^

Although national guidelines have allowed for radiotherapy omission for older patients with low-risk, early-stage BC since 2004, routine omission of adjuvant radiotherapy has not gained traction in the United States. Historical data suggest that up to 65 % of patients who meet criteria for omission of radiotherapy receive this treatment.^5^

The persistent use of adjuvant radiotherapy in patients who are candidates for omission may be driven by recent advances that have improved the convenience of receiving radiotherapy with relatively low morbidity. For example, patients enrolled in the CALGB 9343 trial received conventionally fractionated (CF) whole-breast radiotherapy, which was delivered during 25 daily fractions followed by a seven-fraction boost to the tumor bed, resulting in approximately 6 weeks of treatment.^6^ However, since that time, clinical trials have demonstrated the safety and oncologic equivalence of moderate hypofractionation (MHF) with whole-breast radiotherapy delivered during 15 to 16 daily fractions, ultra-hypofractionation (UHF) with radiotherapy delivered during just 5 fractions, and accelerated partial-breast irradiation (PB) as alternatives to CF whole-breast radiotherapy.^7–10^

Thus, the decision to receive adjuvant radiotherapy is no longer “all” or “none,” and multiple opportunities exist for de-escalation. For older patients who are otherwise candidates for radiotherapy omission but opt for radiotherapy, modern abbreviated regimens (e.g., PB or UHF) have been shown to provide excellent local disease control and to be well-tolerated, convenient, and cost-effective, particularly compared with CF whole-breast radiotherapy.^11,12^ Given equivalent survival and cancer outcomes, treatment with extended courses of radiotherapy for older patients with low-risk disease likely represents overtreatment and warrants substitution with either radiotherapy omission or shorter-course regimens. However, prior studies have historically shown slow adaptation of new radiotherapy regimens in the United States.^13,14^ For example, one study using the National Cancer Database (NCDB) found that in 2016, only 38 % of patients in the United States received hypofractionated whole-breast radiotherapy. In comparison, 70 % to 80 % of patients treated in Canada and the United Kingdom were receiving hypofractionated regimens by 2013.^15,16^

As practice patterns continue to evolve, an updated evaluation of the role of radiotherapy omission for older patients with low-risk BC is needed. The objective of this study was (1) to determine contemporary trends in post-lumpectomy radiotherapy omission for women ≥70 years old with cT1, cN0, ER+/HER2– invasive BC, (2) to determine trends in use of PB, UHF, MHF, and CF radiotherapy regimens for patients who are candidates for radiotherapy omission, and (3) to evaluate patient, tumor, and facility-level factors that contribute to these treatment choices in order to identify potential targets for reducing overtreatment of low-risk BC in the United States.

## Methods

Data were obtained from the NCDB, a registry of cancer cases from more than 1500 facilities accredited by the Commission on Cancer (CoC) in the United States. The NCDB includes data on more than 80 % of all BC cases in the United States.^17^ We identified patients with a diagnosis of invasive BC from 2012 to 2021. The study included patients *≥*70 years old with ER+/HER2–, pathologic T1, clinically node-negative (cN0) invasive BC without distant metastases who underwent lumpectomy, as well as patients who did not undergo surgical axillary staging (pathologic NX). However, patients were excluded if they had pathologically node-positive disease (pN+). Individuals who received any neoadjuvant therapy or adjuvant chemotherapy and those who had distant metastatic disease, had clinical T3/4 tumors, and/or received radiation to any sites other than the breast (i.e., regional nodal irradiation) were excluded. Patients missing tumor receptor status, staging, grade, radiotherapy, or surgical data were excluded. Given the de-identified nature of the data, the study was deemed exempt by the Institutional Review Board.

The NCDB provides data on whether patients received radiotherapy, breast volume (partial- vs whole-breast), and number of fractions during three possible phases of treatment. Patients were defined as having received whole-breast radiotherapy if they received treatment to the whole breast during any phase of treatment with or without PB radiotherapy, PB if they received treatment to the partial breast without whole-breast radiotherapy, and no radiotherapy if they did not receive radiotherapy during any phase of treatment. For patients who received whole-breast radiotherapy, the total number of fractions across all phases of treatment was combined. Based on the total number of treatments, patients were divided into three groups: (1) those receiving 1 to 14 fractions, a proxy for UHF treatment; (2) those receiving 15 to 24 fractions, a proxy for MHF treatment; and (3) those receiving *≥*25 fractions, a proxy for CF treatment. Hospital volume comprising the total number of BC patients per facility during the duration of the study period was defined as low (<1480), medium (1480–2980), and high (>2980), based on previously established definitions.^18^

Patient characteristics were stratified by radiation group, presented as frequency and percentage, and compared across strata using chi-square tests. Trends in adjuvant radiotherapy were presented as frequency and percentage by year within each treatment group; the Mann-Kendall test was performed to assess whether a monotonic trend existed over time within each group. These trends were examined for the overall cohort and within age cohorts. A multivariable logistic regression model was fitted to evaluate the associations of patient characteristics with the receipt of radiotherapy versus radiotherapy omission. A second multivariable logistic regression model was fitted to evaluate the associations of patient characteristics with receipt of CF versus PB, UHF, or MHF radiotherapy regimens. Patients with radiotherapy omission were excluded from this model. Diagnosis year was included in the multivariable models to account for changing clinical practices over time and potential COVID-19 effects in 2020 and 2021. Odds ratios (ORs) and 95 % confidence intervals (CIs) were reported from uni- and multivariable models for each patient characteristic. The *p* values from Wald tests on individual coefficients as well as the overall (type 3) effect of each variable on each radiotherapy outcome were reported. No adjustments were made for multiple comparisons. All statistical analyses were conducted using R, version 4.2.2 (R Foundation for Statistical Computing, Vienna, Austria).

## Results

### Overall Characteristics

The analysis included 121,160 patients who met the criteria. The median age was 75 years (range, 72–80 years), and most of the patients were non-Hispanic white (87.9 %) with low Charlson-Deyo comorbidity scores (76.1 % had a score of 0 and 15.7 % had a score of 1). The majority of the patients had grade 1 or 2 tumors, with only 6.4 % of the patients in the entire cohort having a grade 3 tumor. Whereas 77 % percent of the patients had tumors with ductal histology, 17.9 % had tumors with lobular histology. Surgical nodal staging was performed for 77 % percent of the patients. Notably, 67.7 % of the patients who did not receive radiotherapy underwent axillary staging, compared with 87.3 % of the patients who did receive radiotherapy. The majority (79.3 %) of the patients received endocrine therapy. Overall, 41.9 % of the patients received treatment with both radiotherapy and endocrine therapy, 7.2 % received treatment with radiotherapy alone, 37.4 % received treatment with endocrine therapy alone, and 13.6 % received neither endocrine therapy nor radiotherapy. Most of the patients were treated in comprehensive community cancer programs (43.2 %) or academic/research programs (27.1 %), with 24 %, 29.5 %, and 46.7 % of the patients treated in low-, medium-, and high-volume hospitals, respectively.

Overall, 49.0 % of the patients received adjuvant radiotherapy. Of the patients undergoing radiotherapy, 17.0 % received PB treatment, 8.4 % received UHF whole-breast treatment, 61.7 % received MHF whole-breast treatment, and 12.9 % received CF whole-breast treatment. Full characteristics of the included cohort are shown in Tables 1 and S1.

**Table 1:** Patient characteristics of patients aged *≥*70 years old diagnosed from 2012–2021 in the National Cancer Database with pT1, cN0, ER+/HER2– invasive breast cancer who underwent lumpectomy, stratified by adjuvant radiotherapy regimen

Variable	Overall (*n* = 121,160) *n* (%)	None (*n* = 61,758) *n* (%)	PB (*n* = 10,118) *n* (%)	UHF (*n* = 5003) *n* (%)	MHF (*n* = 36,627) *n* (%)	CF (*n* = 7654) *n* (%)	*p* Value^a^
Age group (years)							<0.001
70–74	52,467 (43.3)	19,776 (32.0)	5519 (54.5)	2666 (53.3)	20,181 (55.1)	4325 (56.5)	
75–79	36,511 (30.1)	18,629 (30.2)	2995 (29.6)	1442 (28.8)	11,119 (30.4)	2326 (30.4)	
80–84	20,854 (17.2)	13,975 (22.6)	1252 (12.4)	664 (13.3)	4166 (11.4)	797 (10.4)	
85+	11,328 (9.3)	9378 (15.2)	352 (3.5)	231 (4.6)	1161 (3.2)	206 (2.7)	
Sex							0.114
Female	120,842 (99.7)	61,575 (99.7)	10,103 (99.9)	4989 (99.7)	36,541 (99.8)	7634 (99.7)	
Male	318 (0.3)	183 (0.3)	15 (0.1)	14 (0.3)	86 (0.2)	20 (0.3)	
Race and ethnicity							<0.001
Hispanic	3832 (3.2)	1803 (2.9)	331 (3.3)	144 (2.9)	1274 (3.5)	280 (3.7)	
NH Asian	2558 (2.1)	1099 (1.8)	202 (2.0)	120 (2.4)	1013 (2.8)	124 (1.6)	
NH black	7435 (6.1)	3911 (6.3)	549 (5.4)	346 (6.9)	2041 (5.6)	588 (7.7)	
NH white	106,527 (87.9)	54,511 (88.3)	8993 (88.9)	4347 (86.9)	32,052 (87.5)	6624 (86.5)	
Other	808 (0.7)	434 (0.7)	43 (0.4)	46 (0.9)	247 (0.7)	38 (0.5)	
Charlson-Deyo Comorbidity Score							<0.001
0	92,146 (76.1)	45,827 (74.2)	7877 (77.9)	3912 (78.2)	28,683 (78.3)	5847 (76.4)	
1	19,042 (15.7)	10,136 (16.4)	1521 (15.0)	710 (14.2)	5366 (14.7)	1309 (17.1)	
2+	9972 (8.2)	5795 (9.4)	720 (7.1)	381 (7.6)	2578 (7.0)	498 (6.5)	
Histology							<0.001
Ductal	92,672 (76.5)	47,052 (76.2)	8165 (80.7)	3971 (79.4)	27,701 (75.6)	5783 (75.6)	
Lobular	21,657 (17.9)	10,735 (17.4)	1435 (14.2)	758 (15.2)	7222 (19.7)	1507 (19.7)	
Other	6831 (5.6)	3971 (6.4)	518 (5.1)	274 (5.5)	1704 (4.7)	364 (4.8)	
Progesterone receptor							<0.001
PR–	11,886 (9.8)	5908 (9.6)	937 (9.3)	474 (9.5)	3824 (10.4)	743 (9.7)	
PR+	109,274 (90.2)	55,850 (90.4)	9181 (90.7)	4529 (90.5)	32,803 (89.6)	6911 (90.3)	
Clinical T category							<0.001
cT0/is	2896 (2.4)	1341 (2.2)	199 (2.0)	103 (2.1)	1065 (2.9)	188 (2.5)	
cT1	114,319 (94.4)	58,521 (94.8)	9635 (95.2)	4772 (95.4)	34,210 (93.4)	7181 (93.8)	
cT2	3945 (3.3)	1896 (3.1)	284 (2.8)	128 (2.6)	1352 (3.7)	285 (3.7)	
Pathologic N category							<0.001
pNX	27,350 (22.6)	19,974 (32.3)	1199 (11.9)	795 (15.9)	4794 (13.1)	588 (7.7)	
pN0	93,810 (77.4)	41,784 (67.7)	8919 (88.1)	4208 (84.1)	31,833 (86.9)	7066 (92.3)	
Grade							<0.001
1	51,377 (42.4)	27,789 (45.0)	4335 (42.8)	2171 (43.4)	14,279 (39.0)	2803 (36.6)	
2	61,981 (51.2)	30,649 (49.6)	5160 (51.0)	2503 (50.0)	19,515 (53.3)	4154 (54.3)	
3	7802 (6.4)	3320 (5.4)	623 (6.2)	329 (6.6)	2833 (7.7)	697 (9.1)	
Facility location							<0.001
Midwest	31,701 (26.2)	16,167 (26.2)	2583 (25.5)	1327 (26.5)	9988 (27.3)	1636 (21.4)	
Northeast	26,428 (21.8)	13,085 (21.2)	2061 (20.4)	1086 (21.7)	8672 (23.7)	1524 (19.9)	
South	40,280 (33.2)	21,573 (34.9)	3280 (32.4)	1599 (32.0)	10,922 (29.8)	2906 (38.0)	
West	22,751 (18.8)	10,933 (17.7)	2194 (21.7)	991 (19.8)	7045 (19.2)	1588 (20.7)	
Facility type							<0.001
Academic/research program	32,825 (27.1)	17,157 (27.8)	3048 (30.1)	1966 (39.3)	9098 (24.8)	1556 (20.3)	
Community cancer program	8809 (7.3)	4545 (7.4)	543 (5.4)	276 (5.5)	2699 (7.4)	746 (9.7)	
Comprehensive community cancer program	52,386 (43.2)	25,947 (42.0)	4288 (42.4)	1768 (35.3)	16,578 (45.3)	3805 (49.7)	
Integrated Network Cancer Program	27,140 (22.4)	14,109 (22.8)	2239 (22.1)	993 (19.8)	8252 (22.5)	1547 (20.2)	
Hospital volume							<0.001
Low (<1480)	28,897 (23.9)	14,682 (23.8)	1882 (18.6)	923 (18.4)	9104 (24.9)	2306 (30.1)	
Medium (1480–2980)	35,705 (29.5)	17,548 (28.4)	3203 (31.7)	1493 (29.8)	10,900 (29.8)	2561 (33.5)	
High (>2980)	56,558 (46.7)	29,528 (47.8)	5033 (49.7)	2587 (51.7)	16,623 (45.4)	2787 (36.4)	
Insurance							<0.001
None	250 (0.2)	119 (0.2)	15 (0.1)	10 (0.2)	86 (0.2)	20 (0.3)	
Medicare	108,116 (89.2)	55,570 (90.0)	9038 (89.3)	4377 (87.5)	32,456 (88.6)	6675 (87.2)	
Medicaid	1319 (1.1)	636 (1.0)	111 (1.1)	39 (0.8)	436 (1.2)	97 (1.3)	
Other government	590 (0.5)	301 (0.5)	47 (0.5)	28 (0.6)	175 (0.5)	39 (0.5)	
Private	10,885 (9.0)	5132 (8.3)	907 (9.0)	549 (11.0)	3474 (9.5)	823 (10.8)	<0.001
Surgical axillary staging	93,810 (77.4)	41,784 (67.7)	8919 (88.1)	4208 (84.1)	31,833 (86.9)	7066 (92.3)	<0.001
Endocrine therapy	96,045 (79.3)	45,339 (73.4)	8330 (82.3)	4045 (80.9)	31,577 (86.2)	6754 (88.2)	<0.001
Diagnosis year							<0.001
2012	5779 (4.8)	3103 (5.0)	521 (5.1)	219 (4.4)	949 (2.6)	987 (12.9)	
2013	6975 (5.8)	3817 (6.2)	617 (6.1)	257 (5.1)	1322 (3.6)	962 (12.6)	
2014	8048 (6.6)	4581 (7.4)	583 (5.8)	269 (5.4)	1659 (4.5)	956 (12.5)	
2015	9325 (7.7)	5399 (8.7)	617 (6.1)	319 (6.4)	2223 (6.1)	767 (10.0)	
2016	9783 (8.1)	5438 (8.8)	594 (5.9)	399 (8.0)	2551 (7.0)	801 (10.5)	
2017	11,330 (9.4)	6175 (10.0)	726 (7.2)	472 (9.4)	3309 (9.0)	648 (8.5)	
2018	16,333 (13.5)	7983 (12.9)	1395 (13.8)	397 (7.9)	5641 (15.4)	917 (12.0)	
2019	18,635 (15.4)	9079 (14.7)	1539 (15.2)	451 (9.0)	6827 (18.6)	739 (9.7)	
2020	15,578 (12.9)	7126 (11.5)	1466 (14.5)	721 (14.4)	5765 (15.7)	500 (6.5)	
2021	19,374 (16.0)	9057 (14.7)	2060 (20.4)	1499 (30.0)	6381 (17.4)	377 (4.9)	

ER, estrogen receptor; HER2, human epidermal growth factor receptor 2; None, no radiation (none); PB, partial-breast; UHF, ultra-hypofractionated whole-breast; MHF, moderately hypofractionated whole-breast; CF, conventionally fractionated whole-breast; NH, non-Hispanic; PR, progesterone receptor

^a^p Values from chi-square tests for categorical variables across strata

### Trends in Adjuvant Radiotherapy

**Fig. 1:**
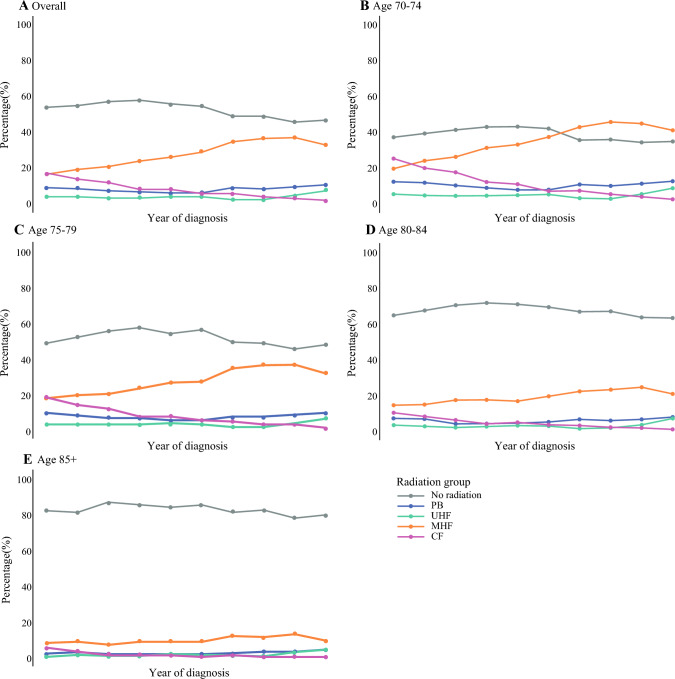
Radiation patterns by year for patients *≥*70 years old who had a diagnosis from 2012 to 2021 in the National Cancer Database with pT1, cN0, ER+/HER2– invasive breast cancer who underwent lumpectomy, stratified by adjuvant radiotherapy regimen: no radiation or partial-breast (PB), ultra-hypofractionated whole-breast (UHF), moderately hypofractionated whole-breast (MHF), or conventionally fractionated whole-breast (CF) radiation. **A** Overall cohort. **B** Age 70–74 years. **C** Age 75–79 years, **D** Age 80–84 years. **E** Age 85+ years. ER, estrogen receptor; HER2, human epidermal growth factor receptor 2

From 2012 to 2021, there were substantial changes in the patterns of radiotherapy receipt (Fig. 1). Initially, radiotherapy omission increased from 53.7 % in 2012 to 57.9 % in 2015. However, from 2016 to 2021, the rates of radiotherapy omission steadily declined, from 55.6 % in 2016 to 46.8 % in 2021 (*p* = 0.032). During this period, the percentage of patients who received MHF whole-breast radiotherapy increased significantly, from 16.4 % in 2012 to 37.0 % in 2020, followed by a slight decrease to 32.9 % in 2021 (*p* < 0.001). In contrast, the cohort of patients who received CF whole-breast radiotherapy decreased significantly, from 17.1 % in 2012 to only 2.0 % in 2021 (*p* < 0.001). The percentage of patients receiving PB treatment did not change (9.0 % in 2012 vs 10.6 % in 2021; *p* = 0.591). Although no overall change in the use of UHF radiotherapy occurred during the study period, from 2012 to 2021, the rates increased sharply by two thirds from 2020 to 2021 (3.8 % in 2012 to 4.6 % in 2020 vs 7.7 % in 2021; *p* = 0.474). In a subset by patient age, the majority of the patients 80 to 84 years old (67.0 %) and those 85 years old (82.8 %) did not receive radiotherapy. This was stable from 2012 to 2021 (*p* = 0.152 to 0.211). In comparison, only 34.9 % of the patients age 70 to 74 years did not receive radiotherapy in 2021. The decline in CF use was of the greatest magnitude among the patients 70 to 74 years old, decreasing from 25.3 % in 2012 to 2.6 % in 2021 (*p* < 0.001).

### Characteristics of Patients Receiving Adjuvant Radiotherapy Versus Radiotherapy Omission

In the multivariable analysis, patients who were older (OR, 0.20–0.62; 95 % CI, 0.19–0.64 for ages 75 to 85+ years vs ages 70 to 74 years) or had higher Charlson-Deyo comorbidity scores (OR, 0.77–0.89; 95 % CI, 0.73–0.92 for scores 1 to 2+ vs score 0) showed lower odds of receiving adjuvant radiotherapy of any regimen than those who did not receive radiotherapy (Table 2). Hispanic patients (OR, 1.08; 95 % CI, 1.01–1.16) and non-Hispanic Asian patients (OR, 1.27; 95 % CI, 1.17–1.39) were more likely to receive adjuvant radiotherapy, whereas patients of other race and ethnicity groups (OR, 0.78; 95 % CI, 0.67–0.90) were less likely to receive radiotherapy than non-Hispanic white patients. Patients with progesterone receptor (PR)-negative tumors (OR, 1.07; 95 % CI, 1.03–1.12 vs PR-positive tumors), higher-grade tumors (OR, 1.20–1.74; 95 % CI, 1.17–1.83 for grade 2 or 3 vs grade 1), larger clinical T category (OR, 1.14; 95 % CI, 1.06–1.22 for cT2 vs cT1), and/or lobular histology (OR, 1.06; 95 % CI, 1.03–1.10 vs ductal) were more likely to receive adjuvant radiotherapy, whereas patients with other non-ductal/non-lobular histology (OR, 0.89; 95 % CI, 0.84–0.94 vs ductal) were less likely to receive adjuvant radiotherapy. Patients who received surgical axillary staging (OR, 2.75; 95 % CI, 2.66–2.84) and/or endocrine therapy (OR, 1.56; 95 % CI, 1.52–1.61) were more likely to receive adjuvant radiotherapy than those who did not receive these therapies. Patients treated in the Midwest, Northeast, or West (OR, 1.21-1.35; 95% CI, 1.17-1.40) were more likely to receive adjuvant radiotherapy than those treated in the South. Patients treated at comprehensive community cancer programs (OR, 0.89- 0.91; 95% CI, 0.85-0.95) for all other facility types vs comprehensive community cancer programs) were more likely to receive adjuvant radiotherapy. Insurance type had no significant effect on receipt of adjuvant radiotherapy (*p* = 0.742). Compared with 2012, the odds of receiving adjuvant radiotherapy decreased from 2013 to 2017 (OR, 0.73–0.89; 95 % CI, 0.68–0.96), then increased from 2018 to 2021 (OR, 1.06-1.31; 95 % CI, 1.00–1.40).

**Table 2:** Multivariable logistic regression for receipt of any adjuvant radiotherapy versus no radiotherapy for patients age *≥*70 years with a diagnosis from 2012 to 2021 in the National Cancer Database with pT1, cN0, ER+/HER2– invasive breast cancer who underwent lumpectomy

Covariate	No radiation *n* (%)	Any radiation *n* (%)	Univariable OR (95 % CI)	Multivariable OR (95 % CI)	Overall (type III) test *p* Value
Age group					
70–74	19776 (37.7)	32691 (62.3)	Ref	Ref	<0.001
75–79	18629 (51.0)	17882 (49.0)	0.58 (0.57–0.60; *p* < 0.001)	0.62 (0.61–0.64; *p* < 0.001)	
80–84	13975 (67.0)	6879 (33.0)	0.30 (0.29–0.31; *p* < 0.001)	0.37 (0.36–0.38; *p* < 0.001)	
85+	9378 (82.8)	1950 (17.2)	0.13 (0.12–0.13; *p* < 0.001)	0.20 (0.19–0.21; *p* < 0.001)	
Race and ethnicity					<0.001
Non-Hispanic white	54511 (51.2)	52016 (48.8)	Ref	Ref	
Hispanic	1803 (47.1)	2029 (52.9)	1.18 (1.11–1.26; *p* < 0.001)	1.08 (1.01–1.16; *p* = 0.035)	
Non-Hispanic Asian	1099 (43.0)	1459 (57.0)	1.39 (1.29–1.51; *p* < 0.001)	1.27 (1.17–1.39; *p* < 0.001)	
Non-Hispanic black	3911 (52.6)	3524 (47.4)	0.94 (0.90–0.99; *p* = 0.017)	0.97 (0.92–1.02; *p* = 0.210)	
Other	434 (53.7)	374 (46.3)	0.90 (0.79–1.04; *p* = 0.150)	0.78 (0.67–0.90; *p* = 0.001)	
Charlson-Deyo Comorbidity Score					<0.001
0	45827 (49.7)	46319 (50.3)	Ref	Ref	
1	10136 (53.2)	8906 (46.8)	0.87 (0.84–0.90; *p* < 0.001)	0.89 (0.86–0.92; *p* < 0.001)	
2+	5795 (58.1)	4177 (41.9)	0.71 (0.68–0.74; *p* < 0.001)	0.77 (0.73–0.80; *p* < 0.001)	
Histology					<0.001
Ductal	47052 (50.8)	45620 (49.2)	Ref	Ref	
Lobular	10735 (49.6)	10922 (50.4)	1.05 (1.02–1.08; *p* = 0.001)	1.06 (1.03–1.10; *p* < 0.001)	
Other	3971 (58.1)	2860 (41.9)	0.74 (0.71–0.78; *p* < 0.001)	0.89 (0.84–0.94; *p* < 0.001)	
Progesterone receptor					<0.001
PR+	55850 (51.1)	53424 (48.9)	Ref	Ref	
PR–	5908 (49.7)	5978 (50.3)	1.06 (1.02–1.10; *p* = 0.004)	1.07 (1.03–1.12; *p* = 0.001)	
Clinical T category					<0.001
cT1	58521 (51.2)	55798 (48.8)	Ref	Ref	
cT0/is	1341 (46.3)	1555 (53.7)	1.22 (1.13–1.31; *p* < 0.001)	1.70 (1.56–1.84; *p* < 0.001)	
cT2	1896 (48.1)	2049 (51.9)	1.13 (1.06–1.21; *p* < 0.001)	1.14 (1.06–1.22; *p* < 0.001)	
Grade					<0.001
1	27789 (54.1)	23588 (45.9)	Ref	Ref	
2	30649 (49.4)	31332 (50.6)	1.20 (1.18–1.23; *p* < 0.001)	1.20 (1.17–1.23; *p* < 0.001)	
3	3320 (42.6)	4482 (57.4)	1.59 (1.52–1.67; *p* < 0.001)	1.74 (1.65–1.83; *p* < 0.001)	
Facility location					<0.001
South	21573 (53.6)	18707 (46.4)	Ref	Ref	
Midwest	16167 (51.0)	15534 (49.0)	1.11 (1.08–1.14; *p* < 0.001)	1.21 (1.17–1.25; *p* < 0.001)	
Northeast	13085 (49.5)	13343 (50.5)	1.18 (1.14–1.21; *p* < 0.001)	1.35 (1.30–1.40; *p* < 0.001)	
West	10933 (48.1)	11818 (51.9)	1.25 (1.21–1.29; *p* < 0.001)	1.34 (1.29–1.38; *p* < 0.001)	
Facility type					<0.001
Comprehensive community cancer program	25947 (49.5)	26439 (50.5)	Ref	Ref	
Academic/research program	17157 (52.3)	15668 (47.7)	0.90 (0.87–0.92; *p* < 0.001)	0.89 (0.86–0.92; *p* < 0.001)	
Community cancer program	4545 (51.6)	4264 (48.4)	0.92 (0.88–0.96; *p* < 0.001)	0.90 (0.85–0.95; *p* < 0.001)	
Integrated Network Cancer Program	14109 (52.0)	13031 (48.0)	0.91 (0.88–0.93; *p* < 0.001)	0.91 (0.88–0.94; *p* < 0.001)	
Hospital volume					<0.001
Low (<1480)	14682 (50.8)	14215 (49.2)	Ref	Ref	
Medium (1480–2980)	17548 (49.1)	18157 (50.9)	1.07 (1.04–1.10; *p* < 0.001)	1.06 (1.02–1.10; *p* = 0.002)	
High (>2980)	29528 (52.2)	27030 (47.8)	0.95 (0.92–0.97; *p* < 0.001)	0.98 (0.94–1.01; *p* = 0.234)	
Insurance					0.742
Government	56507 (51.4)	53518 (48.6)	Ref	Ref	
None	119 (47.6)	131 (52.4)	1.16 (0.91–1.49; *p* = 0.235)	1.14 (0.87–1.49; *p* = 0.335)	
Private	5132 (47.1)	5753 (52.9)	1.18 (1.14–1.23; *p* < 0.001)	1.02 (0.98–1.07; *p* = 0.272)	
Surgical axillary staging					< 0.001
No	19974 (73.0)	7376 (27.0)	Ref	Ref	
Yes	41784 (44.5)	52026 (55.5)	3.37 (3.27–3.47; *p* < 0.001)	2.75 (2.66–2.84; *p* < 0.001)	
Endocrine therapy					<0.001
No	16419 (65.4)	8696 (34.6)	Ref	Ref	
Yes	45339 (47.2)	50706 (52.8)	2.11 (2.05–2.17; *p* < 0.001)	1.56 (1.52–1.61; *p* < 0.001)	
Diagnosis year					
2012	3103 (53.7)	2676 (46.3)	Ref	Ref	
2013	3817 (54.7)	3158 (45.3)	0.96 (0.89–1.03; *p* = 0.245)	0.89 (0.83–0.96; *p* = 0.003)	
2014	4581 (56.9)	3467 (43.1)	0.88 (0.82–0.94; *p* < 0.001)	0.78 (0.72–0.83; *p* < 0.001)	
2015	5399 (57.9)	3926 (42.1)	0.84 (0.79–0.90; *p* < 0.001)	0.73 (0.68–0.78; *p* < 0.001)	
2016	5438 (55.6)	4345 (44.4)	0.93 (0.87–0.99; *p* = 0.022)	0.75 (0.70–0.81; *p* < 0.001)	
2017	6175 (54.5)	5155 (45.5)	0.97 (0.91–1.03; *p* = 0.316)	0.78 (0.73–0.83; *p* < 0.001)	
2018	7983 (48.9)	8350 (51.1)	1.21 (1.14–1.29; *p* < 0.001)	1.06 (1.00–1.14; *p* = 0.060)	
2019	9079 (48.7)	9556 (51.3)	1.22 (1.15–1.29; *p* < 0.001)	1.10 (1.03–1.17; *p* = 0.005)	
2020	7126 (45.7)	8452 (54.3)	1.38 (1.29–1.46; *p* < 0.001)	1.24 (1.16–1.33; *p* < 0.001)	
2021	9057 (46.7)	10317 (53.3)	1.32 (1.25–1.40; *p* < 0.001)	1.31 (1.23–1.40; *p* < 0.001)	

ER, estrogen receptor; HER2, human epidermal growth factor receptor 2; OR, odds ratio; CI, confidence interval; PR, progesterone receptor

### Characteristics of Patients Receiving CF Versus All Other Radiotherapy Regimens (PB, MHF Whole-Breast, or UHF Whole-Breast Radiation)

The multivariable analysis of patients receiving adjuvant radiotherapy showed that non-Hispanic black patients versus non-Hispanic white patients (OR, 1.34; 95 % CI, 1.21–1.48), Hispanic patients versus non-Hispanic white patients (OR, 1.20; 95 % CI, 1.05–1.38), and those with higher Charlson/Deyo comorbidity scores versus 0 (OR, 1.13–1.14; 95 % CI, 1.02–1.25) were significantly more likely to receive CF radiotherapy (Table 3). In contrast, non-Hispanic Asian patients versus non-Hispanic white patients (OR, 0.67; 95 % CI, 0.55–0.81) and older patients (OR 0.57–0.92; 95 % CI, 0.49–0.97 for patients 75 to 85+ years old vs patients 70 to 74 years old) were significantly less likely to receive CF radiotherapy. Patients with higher-grade tumors (OR, 1.21-1.51; 95 % CI, 1.15–1.67 for grade 2 to 3 vs grade 1), lobular versus ductal histology (OR, 1.08; 95 % CI, 1.01–1.15), or cT2 vs cT1 tumors (OR, 1.15; 95 % CI, 1.00–1.32) were at significantly higher odds of receiving CF radiotherapy. There was no significant association between receipt of surgical axillary staging (*p* = 0.591), insurance type (*p* = 0.936), or PR status (*p* = 0.278) and the type of radiotherapy received. Patients who received endocrine therapy were at higher odds of receiving CF radiotherapy (OR, 1.38; 95 % CI, 1.28–1.50). Patients treated in the Midwest, Northeast, and West (OR, 0.57–0.70; 95 % CI, 0.53–0.76 vs the South), at academic/research or Integrated Network Cancer Program hospitals (OR, 0.74–0.77; 95 % CI, 0.69–0.82 vs comprehensive community cancer program hospitals), and/or higher-volume hospitals (OR, 0.57–0.79; 95 % CI, 0.53–0.85 for medium- and high-volume vs low-volume hospitals) were significantly less likely to receive CF radiotherapy than other types of radiotherapy. Compared with 2012, the odds of receiving CF radiotherapy progressively decreased each year (OR, 0.05–0.73; 95 % CI, 0.05–0.81).

**Table 3:** Multivariable logistic regression for receipt of conventionally fractionated whole-breast radiotherapy (CF) versus all other radiotherapy regimens (partial-breast [PB], moderately hypofractionated whole-breast [MHF], or ultra-hypofractionated whole-breast [UHF]) for patients age *≥*70 years with a diagnosis from 2012–2021 in the National Cancer Database with pT1, cN0, ER+/HER2– invasive breast cancer who underwent lumpectomy

Covariate	PB, MHF, or UHF *n* (%)	CF *n* (%)	Univariable OR (95 % CI)	Multivariable OR (95 % CI)	Overall (type III) test *p* Value
Age group					<0.001
70–74	28366 (86.8)	4325 (13.2)	Ref	Ref	
75–79	15556 (87.0)	2326 (13.0)	0.98 (0.93–1.04; *p* = 0.479)	0.92 (0.87–0.97; *p* = 0.004)	
80–84	6082 (88.4)	797 (11.6)	0.86 (0.79–0.93; *p* < 0.001)	0.71 (0.65–0.78; *p* < 0.001)	
85+	1744 (89.4)	206 (10.6)	0.77 (0.67–0.90; *p* = 0.001)	0.57 (0.49–0.67; *p* < 0.001)	
Race/ethnicity					<0.001
Non-Hispanic white	45392 (87.3)	6624 (12.7)	Ref	Ref	
Hispanic	1749 (86.2)	280 (13.8)	1.10 (0.96–1.25; *p* = 0.159)	1.20 (1.05–1.38; *p* = 0.008)	
Non-Hispanic Asian	1335 (91.5)	124 (8.5)	0.64 (0.53–0.76; *p* < 0.001)	0.67 (0.55–0.81; *p* < 0.001)	
Non-Hispanic black	2936 (83.3)	588 (16.7)	1.37 (1.25–1.50; *p* < 0.001)	1.34 (1.21–1.48; *p* < 0.001)	
Other	336 (89.8)	38 (10.2)	0.78 (0.54–1.07; *p* = 0.138)	0.92 (0.64–1.30; *p* = 0.662)	
Charlson-Deyo Comorbidity Score					<0.001
0	40472 (87.4)	5847 (12.6)	Ref	Ref	
1	7597 (85.3)	1309 (14.7)	1.19 (1.12–1.27; *p* < 0.001)	1.14 (1.07–1.22; *p* < 0.001)	
2+	3679 (88.1)	498 (11.9)	0.94 (0.85–1.03; *p* = 0.191)	1.13 (1.02–1.25; *p* = 0.020)	
Histology					0.078
Ductal	39837 (87.3)	5783 (12.7)	Ref	Ref	
Lobular	9415 (86.2)	1507 (13.8)	1.10 (1.04–1.17; *p* = 0.002)	1.08 (1.01–1.15; *p* = 0.025)	
Other	2496 (87.3)	364 (12.7)	1.00 (0.90–1.12; *p* = 0.937)	0.99 (0.88–1.12; *p* = 0.934)	
Progesterone receptor					0.278
PR+	46513 (87.1)	6911 (12.9)	Ref	Ref	
PR–	5235 (87.6)	743 (12.4)	0.96 (0.88–1.04; *p* = 0.267)	0.95 (0.87–1.04; *p* = 0.278)	
Clinical T category					0.006
cT1	48617 (87.1)	7181 (12.9)	Ref	Ref	
cT0/is	1367 (87.9)	188 (12.1)	0.93 (0.80–1.08; *p* = 0.365)	1.23 (1.04–1.45; *p* = 0.013)	
cT2	1764 (86.1)	285 (13.9)	1.09 (0.96–1.24; *p* = 0.168)	1.15 (1.00–1.32; *p* = 0.041)	
Grade					<0.001
1	20785 (88.1)	2803 (11.9)	Ref	Ref	
2	27178 (86.7)	4154 (13.3)	1.13 (1.08–1.19; *p* < 0.001)	1.21 (1.15–1.28; *p* < 0.001)	
3	3785 (84.4)	697 (15.6)	1.37 (1.25–1.49; *p* < 0.001)	1.51 (1.37–1.67; *p* < 0.001)	
Facility location					<0.001
South	15801 (84.5)	2906 (15.5)	Ref	Ref	
Midwest	13898 (89.5)	1636 (10.5)	0.64 (0.60–0.68; *p* < 0.001)	0.57 (0.53–0.61; *p* < 0.001)	
Northeast	11819 (88.6)	1524 (11.4)	0.70 (0.66–0.75; *p* < 0.001)	0.68 (0.63–0.73; *p* < 0.001)	
West	10230 (86.6)	1588 (13.4)	0.84 (0.79–0.90; *p* < 0.001)	0.70 (0.66–0.76; *p* < 0.001)	
Facility type					<0.001
Comprehensive community cancer program	22634 (85.6)	3805 (14.4)	Ref	Ref	
Academic/research program	14112 (90.1)	1556 (9.9)	0.66 (0.62–0.70; *p* < 0.001)	0.74 (0.69–0.79; *p* < 0.001)	
Community cancer program	3518 (82.5)	746 (17.5)	1.26 (1.16–1.37; *p* < 0.001)	1.07 (0.96–1.18; *p* = 0.209)	
Integrated Network Cancer Program	11484 (88.1)	1547 (11.9)	0.80 (0.75–0.85; *p* < 0.001)	0.77 (0.72–0.82; *p* < 0.001)	
Hospital volume					<0.001
Low (<1480)	11909 (83.8)	2306 (16.2)	Ref	Ref	
Medium (1480–2980)	15596 (85.9)	2561 (14.1)	0.85 (0.80–0.90; *p* < 0.001)	0.79 (0.73–0.85; *p* < 0.001)	
High (>2980)	24243 (89.7)	2787 (10.3)	0.59 (0.56–0.63; *p* < 0.001)	0.57 (0.53–0.62; *p* < 0.001)	
Insurance					0.936
Government	46707 (87.3)	6811 (12.7)	Ref	Ref	
None	111 (84.7)	20 (15.3)	1.24 (0.75–1.94; *p* = 0.384)	1.05 (0.62–1.71; *p* = 0.851)	
Private	4930 (85.7)	823 (14.3)	1.14 (1.06–1.24; *p* = 0.001)	0.99 (0.91–1.07; *p* = 0.757)	
Surgical axillary staging					0.591
No	6788 (92.0)	588 (8.0)	Ref	Ref	
Yes	44960 (86.4)	7066 (13.6)	1.81 (1.66–1.98; *p* < 0.001)	0.97 (0.88–1.07; *p* = 0.591)	
Endocrine therapy					<0.001
No	7796 (89.7)	900 (10.3)	Ref	Ref	
Yes	43952 (86.7)	6754 (13.3)	1.33 (1.24–1.43; *p* < 0.001)	1.38 (1.28–1.50; *p* < 0.001)	
Diagnosis year					<0.001
2012	1689 (63.1)	987 (36.9)	Ref	Ref	
2013	2196 (69.5)	962 (30.5)	0.75 (0.67–0.84; *p* < 0.001)	0.73 (0.65–0.81; *p* < 0.001)	
2014	2511 (72.4)	956 (27.6)	0.65 (0.58–0.73; *p* < 0.001)	0.61 (0.55–0.69; *p* < 0.001)	
2015	3159 (80.5)	767 (19.5)	0.42 (0.37–0.46; p < 0.001)	0.39 (0.34–0.43; *p* < 0.001)	
2016	3544 (81.6)	801 (18.4)	0.39 (0.35–0.43; *p* < 0.001)	0.35 (0.31–0.39; *p* < 0.001)	
2017	4507 (87.4)	648 (12.6)	0.25 (0.22–0.28; *p* < 0.001)	0.21 (0.19–0.24; *p* < 0.001)	
2018	7433 (89.0)	917 (11.0)	0.21 (0.19–0.23; *p* < 0.001)	0.18 (0.16–0.20; *p* < 0.001)	
2019	8817 (92.3)	739 (7.7)	0.14 (0.13–0.16; *p* < 0.001)	0.12 (0.11–0.13; *p* < 0.001)	
2020	7952 (94.1)	500 (5.9)	0.11 (0.10–0.12; *p* < 0.001)	0.09 (0.08–0.10; *p* < 0.001)	
2021	9940 (96.3)	377 (3.7)	0.06 (0.06–0.07; *p* < 0.001)	0.05 (0.05–0.06; *p* < 0.001)	

ER, estrogen receptor; HER2, human epidermal growth factor receptor 2; OR, odds ratio; CI, confidence interval; PR, progesterone receptor

## Discussion

Despite long-term data supporting the oncologic safety of omission of post-lumpectomy radiotherapy for patients *≥*70 years old with early-stage, ER+/HER2– invasive BC who receive adjuvant endocrine therapy, approximately half of these patients continue to receive radiotherapy. The persistent use of adjuvant radiotherapy suggests that the lack of practice change is not due to a lag between dissemination and implementation of evidence-based research, but an indication that patients and/or providers continue to identify a role for radiotherapy in these patients. Our results show an initial uptake in radiotherapy omission from 2012 to 2016, which coincides with the release of 10-year data from the CALGB 9343 trial in 2014 and 5-year data from the PRIME II trial in 2015, both demonstrating an improvement in rates of locoregional recurrence but no survival benefit of adjuvant radiotherapy for older patients with low-risk ER+/HER2– BC.^2,19^ This is consistent with findings from prior studies using the Surveillance, Epidemiology, and End Results (SEER) database, which showed a decrease in overtreatment with high-intensity treatment regimens from 2010 to 2015.^20^ However, our study showed a trend reversal in recent years, with a steady decline in radiotherapy omission, from 56 % in 2016 to 47 % in 2021.

One explanation for this finding may be increased availability and acceptability of abbreviated methods for radiotherapy delivery. This is supported by our observation that during the last decade, the percentage of patients who received CF radiotherapy decreased from 17 % in 2012 to only 2.0 % in 2021, whereas the percentage of patients who received MHF radiotherapy doubled. This practice is in line with recommendations from national organizations including the National Comprehensive Cancer Network (NCCN) and the American Society for Therapeutic Radiology and Oncology (ASTRO), which have recommended MHF radiotherapy as the standard of care for low-risk patients since 2011 and for all patients since 2018.^3,21^ Notably, in the last 2 years of our study, the percentage of patients who received MHF treatment decreased from 37.0 % to 32.9 %. However, this decrease was accompanied by a commensurate increase in UHF delivery from 4.6 % to 7.7 %.

Thus, there may be two opportunities to avoid radiotherapy overtreatment of older women with early-stage ER+/HER2– BC: (1) increasing rates of radiotherapy omission or (2) increasing rates of abbreviated radiotherapy regimens for patients who elect to receive adjuvant radiotherapy. To date, most studies on radiotherapy de-escalation have focused on radiotherapy omission. However, these studies have identified multi-level barriers to omitting adjuvant radiotherapy. For example, although efforts have been made to incorporate frailty and geriatric assessments to encourage de-escalated treatment for the patients who are most at risk for overtreatment, these instruments have not been widely integrated into routine clinical care.^22^ Additionally, although adjuvant radiotherapy does not improve survival for older patients with low-risk ER+/HER2– BC, it does significantly decrease the 10-year risk of locoregional recurrence, from approximately 10 % to less than 1 %.^2,4^ This locoregional benefit is influential. When surveyed, older patients stated that peace of mind and a desire to prevent their cancer from returning were significant motivators for their treatment choices.^23^ Finally, providers may not feel that radiotherapy omission is appropriate for some patients with higher-risk tumor features such as close microscopic margins, lymphovascular invasion, and lower ER positivity because those patients may have been underrepresented in the omission trials or have been shown to be at higher risk for locoregional recurrence.^24,25^ This practice trend is reflected in our study because we found that patients with larger, high-grade tumors were significantly more likely to receive adjuvant radiotherapy.

Currently, candidacy for radiotherapy omission requires that patients take adjuvant endocrine therapy for 5 years.^3^ However, studies suggest that 20 % to 50 % of patients discontinue endocrine therapy before 5 years. For these patients, the PRIME II trial demonstrated a fourfold increased risk of locoregional recurrence for those who did not receive radiotherapy.^4,26,27^ However, our study did not show greater use of radiotherapy for patients who omitted endocrine therapy. Rather, the opposite was true; patients who received radiotherapy were significantly more likely to also receive endocrine therapy. This suggests that patients and providers are not currently substituting radiotherapy for endocrine therapy. This may change in the future. A recent study demonstrated that overall survival may be similar for those who elect to receive monotherapy with either adjuvant radiotherapy or endocrine therapy, and maturing data from the EUROPA trial are evaluating the safety of radiotherapy versus endocrine monotherapy in this patient population.^28,29^

Importantly, patients who are undertreated and receive neither endocrine therapy nor radiotherapy are at significant risk of worse cancer outcomes. Studies show worse locoregional recurrence-free, disease-free, and overall survival than for patients who receive at least one adjuvant therapy.^4,29,30^ Thus, providers may be erring on the side of caution and treating patients with adjuvant radiotherapy because it may not be known if a patient will not adhere to endocrine therapy until the window for administering radiotherapy has passed.

Given the barriers to increasing rates of radiotherapy omission, an alternate strategy to avoid overtreatment is to promote de-escalated regimens for patients who elect to receive radiotherapy. The United States has traditionally lagged behind other countries in adopting shorter-course radiotherapy regimens. For example, current National Institute for Health and Care Excellence (NICE) guidelines from the United Kingdom support UHF as the standard of care for whole-breast irradiation.^31^ This recommendation has *not* been adopted in the United States, although this may change in response to a recent presentation of 10-year data from the UK Fast Forward trial supporting the safety of UHF. Promoting de-escalation of radiotherapy regimens is crucial because they offer equivalent cancer outcomes but significant advantages in terms of quality of life and cost.^8–10^ Shorter courses of radiotherapy are generally more cost-effective and more convenient for patients, particularly older patients who may have medical comorbidities and be reliant on caregivers for transportation.^12,32,33^

Our study demonstrated distinct patient-, tumor-, and facility-level characteristics associated with receipt of extended courses of radiotherapy. Younger patients with higher-grade or lobular tumors were more likely to receive 25+ fractions of radiotherapy, which could reflect provider concern that patients with higher-risk tumors are not candidates for hypofractionated radiotherapy regimens. However, current national recommendations do not support this practice pattern, and ASTRO has recommended hypofractionation for these patients since 2018.^21^

Finally, our results showed significantly lower odds of receiving CF for patients treated in academic/research and Integrated Network Cancer Program facilities and in higher-volume facilities, and higher odds of receiving CF among patients with private insurance versus government insurance, suggesting that payment and care delivery models may contribute to selection of radiotherapy treatment methods or are surrogates for socioeconomic factors impacting quality of care. These factors should be evaluated when strategies are designed to promote de-escalation of radiotherapy for older patients with early-stage, ER+/HER2– BC.

Our study was limited by its retrospective nature and at risk for treatment bias and possible data entry issues inherent to national cancer registries. Additionally, there is no variable denoting whether patients received CF, MHF, UHF, or PB irradiation, and these categories were defined by extrapolating from the treatment sites and number of fractions administered. Given the nature of this study, no data on multi-disciplinary influence that may impact treatment decisions are available.

Finally, our study duration encompassed 2020, and it is unclear whether the trends seen in the later years of our study were impacted by the COVID-19 pandemic. Future studies are needed to confirm that the trends observed in our study were not a temporary response to resource restrictions during the COVID-19 pandemic but rather reflect sustained practice changes.

## Conclusion

In summary, our study demonstrated increasing rates of radiotherapy for older patients with low-risk ER+/HER2– BC who are candidates for radiotherapy omission. This finding may be related to increased use of shorter-course radiotherapy regimens during the last decade. However, we propose that further evidence-based de-escalation is warranted to balance the potential benefits of adjuvant therapies against the potential harms of overtreatment. For patients who elect to receive radiotherapy despite the safety of radiotherapy omission, UHF or PB irradiation should be considered the standard of care. Encouragingly, our study suggests that this may already be happening, with a substantial increase in the use of UHF during the last year of our study.

## Supplementary Information

Below is the link to the electronic supplementary material.

Supplementary file1 (DOCX 34 KB)

## Data Availability

All data used for this study are from the NCDB which is freely available to CoC-accredited facilities upon request.
