# Supplementary material for: Radiation De-Escalation in Older Women with Early-Stage ER+/HER2– Invasive Breast Cancer
Source: Ann Surg Oncol. 2025 Nov 23;33(3):2284–97. doi: 10.1245/s10434-025-18746-z (PMC12901127; doi:10.1245/s10434-025-18746-z)
Supplement: Supplementary file 1 — Supplementary file1 (DOCX 34 KB) [file 10434_2025_18746_MOESM1_ESM.docx]

**Table S1.** Patient characteristics of patients aged ≥70 years old diagnosed from 2012-2021 in the National Cancer Database with pT1, cN0, ER+/HER2- invasive breast cancer who underwent lumpectomy, stratified by adjuvant radiotherapy and endocrine therapy receipt.^1^

| **Variable** | **Overall**  **n=121,160** | **ET only**  **n=45,339** | **RT only**  **n=8,696** | **ET and RT**  **n=50,706** | **None**  **n=16,419** | **p-value^2^** |
| --- | --- | --- | --- | --- | --- | --- |
| Age Group |  |  |  |  |  | <0.001 |
| 70-74 | 52467  (43.3%) | 15613  (34.4%) | 3470  (39.9%) | 29221  (57.6%) | 4163  (25.4%) |  |
| 75-79 | 36511  (30.1%) | 14670  (32.4%) | 2738  (31.5%) | 15144  (29.9%) | 3959  (24.1%) |  |
| 80-84 | 20854  (17.2%) | 9974  (22.0%) | 1736  (20.0%) | 5143  (10.1%) | 4001  (24.4%) |  |
| 85+ | 11328  (9.3%) | 5082  (11.2%) | 752  (8.6%) | 1198  (2.4%) | 4296  (26.2%) |  |
| Sex |  |  |  |  |  | <0.001 |
| Female | 120842  (99.7%) | 45231  (99.8%) | 8667  (99.7%) | 50600  (99.8%) | 16344  (99.5%) |  |
| Male | 318  (0.3%) | 108  (0.2%) | 29  (0.3%) | 106  (0.2%) | 75  (0.5%) |  |
| Race and Ethnicity |  |  |  |  |  | <0.001 |
| Hispanic | 3832  (3.2%) | 1232  (2.7%) | 234  (2.7%) | 1795  (3.5%) | 571  (3.5%) |  |
| NH^3^ Asian | 2558  (2.1%) | 834  (1.8%) | 205  (2.4%) | 1254  (2.5%) | 265  (1.6%) |  |
| NH Black | 7435  (6.1%) | 3003  (6.6%) | 409  (4.7%) | 3115  (6.1%) | 908  (5.5%) |  |
| NH White | 106527  (87.9%) | 39967  (88.2%) | 7801  (89.7%) | 44215  (87.2%) | 14544  (88.6%) |  |
| Other | 808  (0.7%) | 303  (0.7%) | 47  (0.5%) | 327  (0.6%) | 131  (0.8%) |  |
| Charlson/Deyo Comorbidity Score |  |  |  |  |  | <0.001 |
| 0 | 92146  (76.1%) | 33591  (74.1%) | 6794  (78.1%) | 39525  (77.9%) | 12236  (74.5%) |  |
| 1 | 19042  (15.7%) | 7529  (16.6%) | 1260  (14.5%) | 7646  (15.1%) | 2607  (15.9%) |  |
| 2+ | 9972  (8.2%) | 4219  (9.3%) | 642  (7.4%) | 3535  (7.0%) | 1576  (9.6%) |  |
| Histology |  |  |  |  |  | <0.001 |
| Ductal | 92672  (76.5%) | 34656  (76.4%) | 6669  (76.7%) | 38951  (76.8%) | 12396  (75.5%) |  |
| Lobular | 21657  (17.9%) | 7981  (17.6%) | 1445  (16.6%) | 9477  (18.7%) | 2754  (16.8%) |  |
| Other | 6831  (5.6%) | 2702  (6.0%) | 582  (6.7%) | 2278  (4.5%) | 1269  (7.7%) |  |
| Progesterone Receptor (PR) |  |  |  |  |  | <0.001 |
| PR- | 11886  (9.8%) | 4216  (9.3%) | 991  (11.4%) | 4987  (9.8%) | 1692  (10.3%) |  |
| PR+ | 109274  (90.2%) | 41123  (90.7%) | 7705  (88.6%) | 45719  (90.2%) | 14727  (89.7%) |  |
| Clinical T-category |  |  |  |  |  | <0.001 |
| cT0/is | 2896  (2.4%) | 883  (1.9%) | 280  (3.2%) | 1275  (2.5%) | 458  (2.8%) |  |
| cT1 | 114319  (94.4%) | 43108  (95.1%) | 8172  (94.0%) | 47626  (93.9%) | 15413  (93.9%) |  |
| cT2 | 3945  (3.3%) | 1348  (3.0%) | 244  (2.8%) | 1805  (3.6%) | 548  (3.3%) |  |
| Pathological N-category |  |  |  |  |  | <0.001 |
| pNX | 27350  (22.6%) | 13563  (29.9%) | 1745  (20.1%) | 5631  (11.1%) | 6411  (39.0%) |  |
| pN0 | 93810  (77.4%) | 31776  (70.1%) | 6951  (79.9%) | 45075  (88.9%) | 10008  (61.0%) |  |
| Grade |  |  |  |  |  | <0.001 |
| 1 | 51377  (42.4%) | 20176  (44.5%) | 4101  (47.2%) | 19487  (38.4%) | 7613  (46.4%) |  |
| 2 | 61981  (51.2%) | 22782  (50.2%) | 4056  (46.6%) | 27276  (53.8%) | 7867  (47.9%) |  |
| 3 | 7802  (6.4%) | 2381  (5.3%) | 539  (6.2%) | 3943  (7.8%) | 939  (5.7%) |  |
| Facility Location |  |  |  |  |  | <0.001 |
| Midwest | 31701  (26.2%) | 12516  (27.6%) | 2072  (23.8%) | 13462  (26.5%) | 3651  (22.2%) |  |
| Northeast | 26428  (21.8%) | 9832  (21.7%) | 1925  (22.1%) | 11418  (22.5%) | 3253  (19.8%) |  |
| South | 40280  (33.2%) | 15652  (34.5%) | 2376  (27.3%) | 16331  (32.2%) | 5921  (36.1%) |  |
| West | 22751  (18.8%) | 7339  (16.2%) | 2323  (26.7%) | 9495  (18.7%) | 3594  (21.9%) |  |
| Facility Type |  |  |  |  |  | <0.001 |
| Academic/  Research  Program | 32825  (27.1%) | 13231  (29.2%) | 2246  (25.8%) | 13422  (26.5%) | 3926  (23.9%) |  |
| Community  Cancer  Program | 8809  (7.3%) | 3258  (7.2%) | 572  (6.6%) | 3692  (7.3%) | 1287  (7.8%) |  |
| Comp  Community  Cancer  Program | 52386  (43.2%) | 18518  (40.8%) | 3863  (44.4%) | 22576  (44.5%) | 7429  (45.2%) |  |
| Integrated  Network  Cancer  Program | 27140  (22.4%) | 10332  (22.8%) | 2015  (23.2%) | 11016  (21.7%) | 3777  (23.0%) |  |
| Hospital Volume |  |  |  |  |  | <0.001 |
| Low  (<1480) | 28897  (23.9%) | 10773  (23.8%) | 1861  (21.4%) | 12354  (24.4%) | 3909  (23.8%) |  |
| Medium  (1480-2980) | 35705  (29.5%) | 12680  (28.0%) | 2849  (32.8%) | 15308  (30.2%) | 4868  (29.6%) |  |
| High  (>2980) | 56558  (46.7%) | 21886  (48.3%) | 3986  (45.8%) | 23044  (45.4%) | 7642  (46.5%) |  |
| Insurance |  |  |  |  |  | <0.001 |
| None | 250  (0.2%) | 89  (0.2%) | 14  (0.2%) | 117  (0.2%) | 30  (0.2%) |  |
| Medicare | 108116  (89.2%) | 40931  (90.3%) | 7766  (89.3%) | 44780  (88.3%) | 14639  (89.2%) |  |
| Medicaid | 1319  (1.1%) | 474  (1.0%) | 91  (1.0%) | 592  (1.2%) | 162  (1.0%) |  |
| Other Government | 590  (0.5%) | 228  (0.5%) | 45  (0.5%) | 244  (0.5%) | 73  (0.4%) |  |
| Private | 10885  (9.0%) | 3617  (8.0%) | 780  (9.0%) | 4973  (9.8%) | 1515  (9.2%) |  |
| Surgical Axillary Staging | 93810 (77.4%) | 31776  (70.1%) | 6951  (79.9%) | 45075  (88.9%) | 10008  (61.0%) | <0.001 |
| Diagnosis year |  |  |  |  |  | <0.001 |
| 2012 | 5779  (4.8%) | 1904  (4.2%) | 540  (6.2%) | 2136  (4.2%) | 1199  (7.3%) |  |
| 2013 | 6975  (5.8%) | 2548  (5.6%) | 576  (6.6%) | 2582  (5.1%) | 1269  (7.7%) |  |
| 2014 | 8048  (6.6%) | 3178  (7.0%) | 543  (6.2%) | 2924  (5.8%) | 1403  (8.5%) |  |
| 2015 | 9325  (7.7%) | 3883  (8.6%) | 638  (7.3%) | 3288  (6.5%) | 1516  (9.2%) |  |
| 2016 | 9783  (8.1%) | 4000  (8.8%) | 677  (7.8%) | 3668  (7.2%) | 1438  (8.8%) |  |
| 2017 | 11330  (9.4%) | 4648  (10.3%) | 767  (8.8%) | 4388  (8.7%) | 1527  (9.3%) |  |
| 2018 | 16333  (13.5%) | 5964  (13.2%) | 1133  (13.0%) | 7217  (14.2%) | 2019  (12.3%) |  |
| 2019 | 18635  (15.4%) | 6866  (15.1%) | 1243  (14.3%) | 8313  (16.4%) | 2213  (13.5%) |  |
| 2020 | 15578  (12.9%) | 5550  (12.2%) | 1109  (12.8%) | 7343  (14.5%) | 1576  (9.6%) |  |
| 2021 | 19374  (16.0%) | 6798  (15.0%) | 1470  (16.9%) | 8847  (17.4%) | 2259  (13.8%) |  |
| ^1^Endocrine therapy (ET), radiotherapy (RT).  ^2^p-values from Chi-square tests for categorical variables across strata.  ^3^Non-Hispanic (NH) | | | | | | |
